# Distinct brainstem to spinal cord noradrenergic pathways inversely regulate spinal neuronal activity

**DOI:** 10.1093/brain/awac085

**Published:** 2022-03-04

**Authors:** Mateusz W Kucharczyk, Francesca Di Domenico, Kirsty Bannister

**Affiliations:** Central Modulation of Pain, Institute of Psychiatry, Psychology and Neuroscience, King’s College London, London SE1 1UL, UK; Central Modulation of Pain, Institute of Psychiatry, Psychology and Neuroscience, King’s College London, London SE1 1UL, UK; Central Modulation of Pain, Institute of Psychiatry, Psychology and Neuroscience, King’s College London, London SE1 1UL, UK

**Keywords:** noradrenaline, locus coeruleus, descending modulation, diffuse noxious inhibitory controls, spinal wide dynamic range neurons

## Abstract

Brainstem to spinal cord noradrenergic pathways include a locus coeruleus origin projection and diffuse noxious inhibitory controls. While both pathways are traditionally viewed as exerting an inhibitory effect on spinal neuronal activity, the locus coeruleus was previously shown to have a facilitatory influence on thermal nocioception according to the subpopulation of coerulean neurons activated. Coupled with knowledge of its functional modular organisation and the fact that diffuse noxious inhibitory controls are not expressed in varied animal models of chronicity, we hypothesized a regulatory role for the locus coeruleus on non-coerulean, discrete noradrenergic cell group(s).

We implemented locus coeruleus targeting strategies by microinjecting canine adenovirus encoding for channelrhodopsin-2 under a noradrenaline-specific promoter in the spinal cord (retrogradely labelling a coeruleospinal module) or the locus coeruleus itself (labelling the entire coerulean module). Coeruleospinal module optoactivation abolished diffuse noxious inhibitory controls (two-way ANOVA, *P* < 0.0001), which were still expressed following locus coeruleus neuronal ablation.

We propose that the cerulean system interacts with, but does not directly govern, diffuse noxious inhibitory controls. This mechanism may underlie the role of the locus coeruleus as a ‘chronic pain generator’. Pinpointing the functionality of discrete top-down pathways is crucial for understanding sensorimotor modulation in health and disease.

## Teaser

Nerve pathways project from the brain to the spinal cord to influence pain perception. Kucharczyk *et al.* investigate the relationship between two such pathways and show that their ability to inhibit pain depends on their connectivity, thus revealing a separable function for parallel brain-spine circuits.

## Introduction

The descending pain modulatory system encompasses noradrenergic projections that underpin a tonic pathway from the locus coeruleus (LC) to the dorsal horn of the spinal cord^1^ and diffuse noxious inhibitory controls (DNICs).^2^ Previously, upon (i) activation of the DNIC pathway; or (ii) chemogenetic activation of descending noradrenergic controls following spinal microinjection of canine adenovirus (CAV), wide dynamic range (WDR) neuronal activity was inhibited in a manner that was reversed by spinal application of the α_2_-adrenoreceptor (AR) antagonist atipamezole.^3,4^ Meanwhile, channelrhodopsin-2 (ChR2)-mediated activation of the LC following direct LC lentivirus microinjection (thus labelling a LC:LC module) caused inhibition of the spinal reflex only when the optic fibre was placed ventrally, whereupon atipamezole no longer reversed the inhibitory effect.^5^ Intriguingly, in the same study, optoactivation of the dorsal LC noradrenergic neuron population had a pain potentiating effect.

Tying the knowledge from these studies together, we hypothesized that either a separate coerulean module operates, via α_2_-ARs, to mediate DNICs or that the LC might have a regulatory function on non-coerulean, discrete noradrenergic cell group(s), for example those from where DNIC originates according to its modular organization.^4–6^

Thus, we aimed to dissect the effects of optogenetic activation of selected LC modules on the mechanically evoked activity of spinal WDR neurons and DNIC expression, while investigating the sub-serving pharmacology. By using CAV-PRS-ChR2 to label an LC:SC or LC:LC circuit, we have shown that (i) the LC-SC circuit operates via spinal α_1_-ARs to cause neuronal inhibition; and (ii) DNIC expression is abolished upon its optogenetic activation. Thus, we propose that LC:SC and DNIC pathways are functionally distinct, which has implications for the pain, as well as broader sensorimotor, field.

## Materials and methods

### Animals

Male Sprague-Dawley rats were used for experiments. All procedures described were approved by the Home Office and adhered to the Animals (Scientific Procedures) Act 1986, International Association for Study of Pain^7^ and ARRIVE guidelines.^8^

All experiments were designed to contain minimum of six rats per group, based on G-power predictions from previous experiments. Animals were randomly assigned to experimental groups. From 60 rats designated for this study, seven failed to provide stable WDR neuronal recordings, three developed vestibular problems reaching humane endpoint within 4 days after LC virus microinjection, and one animal died 24 h after administration of a coerulean noradrenergic neurotoxin [*N*-(2-chloroethyl)-*N*-ethyl-2-bromobenzylamine hydrochloride, DSP4]. In total, 49 rats were used. Twenty-four rats were used for mixed opto-pharmacology experiments (six rats per group: LC:LC atipamezole; LC:LC prazosin; LC:SC atipamezole; and LC:SC prazosin). An additional three rats were used for WDR baseline characterization with optogenetics (two for LC:SC and one for LC:LC), followed by LC optoelectrical recordings of transduced neurons. In the latter, no pharmacology was performed. A further six rats were used for the DSP4 group, and 15 were used as naïve controls, which included six used for the lidocaine microinjection experiment.

### DSP4 injections

For ablation of the coerulean noradrenergic fibres across the neuroaxis, 50 mg/kg of the selective neurotoxin DSP4 (Sigma) were injected intraperitoneally.^9–11^

### Coerulean neuron transduction

To transduce catecholaminergic coerulean neurons, ipsilateral LC stereotaxic injections of CAV carrying ChR2 under the control of a catecholamine-specific synthetic promoter (sPRS)^12^ [CAV-sPRS-hChR2(H134R)-mCherry, titre >3 × 10^10^ TU/ml, produced by Plateforme de Vectorologie de Montpellier, France, a gift from Professor Anthony Pickering, University of Bristol^5,6^] were carried out (Kopf Instruments) analogously to that described in detail earlier.^5^ To transduce spinally projecting catecholaminergic brainstem neurons, the same virus was injected in the lumbar spinal cord.^13^

### Spinal cord *in vivo* electrophysiology

*In vivo* electrophysiology was performed on animals weighing 240–300 g as previously described^14^ under isoflurane/N_2_O anaesthesia. Physiological homeostasis was maintained and monitored throughout the experiment. Extracellular single-unit activity of spinal WDR neurons in deep laminae IV/V was measured. Natural mechanical stimuli, including brush and von Frey filaments (8 g, 26 g and 60 g) and von Frey filaments with concurrent ipsilateral noxious ear pinch (to trigger DNIC^3^), were applied in this order to the receptive field for 10 s per stimulus. DNICs are reflected as the inhibitory effect on WDR neuronal firing during ear pinch to its immediate respective von Frey filament applied without the conditioning stimulus (% of inhibition after ear pinch). After collection of predrug baseline control, 100 μg atipamezole (an α_2_-AR antagonist) or 20 μg prazosin hydrochloride (an α_1_-AR antagonist) were administered topically on the spinal cord. All plotted data represent the time point of peak change (10–30 min post application).

### Optogenetics

A simultaneous recording and optical stimulation of the transduced LC neurons were made using microoptrodes as described earlier with minor modifications^15^ to find optimal stimulus parameters. LC neurons were identified as previously described.^5^

The 450 nm laser was used to deliver defined light pulses: 20 ms pulse width at 5 Hz, 30 mW (238 mW/mm^2^) light power density at the tip of the implantable 200 μm fibre cannula.^5^ Spinal WDR neurons were characterized by three stable baseline responses, followed by three optically modulated responses. For combined optogenetics and spinal pharmacology, after collecting three stable baseline and three stable optoactivation responses, a drug was applied topically on the spinal cord surface. At the end of every experiment, animals were sacrificed by the overdose of isoflurane and transcardially perfused with saline followed by 4% paraformaldehyde for anatomical evaluation.

### Lidocaine block of locus coeruleus activity

Six naïve rats were used for lidocaine (500 nl, 2% in saline) block of neuronal activity in the ipsilateral LC during electrophysiological WDR neuron recordings. At the end of the experiment, the solution in the pipette was replaced with 0.5% Lucifer yellow-CH dipotassium salt to mark the injection site.

### Immunohistochemistry

Cryosected tissue was incubated with primary antibodies against dopamine-β-hydroxylase (a marker of noradrenergic neurons: 1:500, Millipore), mCherry (1:500, Abcam), followed by appropriate fluorophore-conjugated secondary antibodies. DAPI was used as nuclear marker. Samples were imaged with an LSM 710 laser-scanning confocal microscope (Zeiss) using Zeiss Plan Achromat 10× (0.3 NA) and 20× (0.8 NA) dry objectives and analysed with Fiji Win 64. For quantification, samples were imaged with a 20× dry objective on a Zeiss Imager Z1 microscope. Six to eight slices were imaged per animal. Cell counting was carried out in Fiji Win 64 using the cell counter plugin. On average, 20–30 brainstem sections were imaged for quantification.

### Passive tissue clearing

A passive CLARITY tissue clearing technique (PACT) was implemented to allow imaging of thick (1000–2500 µm) spinal cord fragments.^9^ Anti-tyrosine hydroxylase (a marker of catecholaminergic neurons; 1:250, Millipore) primary antibody was used, followed by Alexa Fluor 647 secondary antibody (1:200, Invitrogen). After achieving equilibrium with a refractive index-matching solution (refractive index = 1.47), samples were imaged with a Zeiss LSM 780 confocal upright microscope equipped with a Plan-Neofluar 10× (0.3 NA) dry objective and 633 nm laser line. Scans were taken at a resolution of 2048 × 2048 pixels, with a 4–5 μm optical section typically spanning 400–700 μm of scanned depth (resulting in 100–150 planes) with auto Z-brightness correction. Images were analysed with Zen 2012 Blue Edition software followed by Fiji (ImageJ) equipped with appropriate plugins.

### Quantification and statistical analysis

Typically, up to four WDR neurons were characterized per preparation (*n*), and data were collected from at least six rats per group (*N*). A single pharmacological investigation was performed on one neuron per animal. Statistical analysis was performed either on *n* for populational studies or *N* for pharmacological studies. Uncorrected two-way repeated-measures ANOVA with the Tukey *post hoc* test was used to assess von Frey and DNIC responses in the baseline conditions. For the pharmacological experiments, Geisser-Greenhouse correction was used for the repeated-measures ANOVA. A paired student *t*-test was used to assess brush-evoked responses. GraphPad Prism was used to analyse the data. *P* < 0.05 was considered significant.

### Data availability

Data are available upon request.

## Results

### A ventral coerulean neuronal population inhibits spinal nocioceptive processing via α1-adrenoceptors

**Figure 1:**
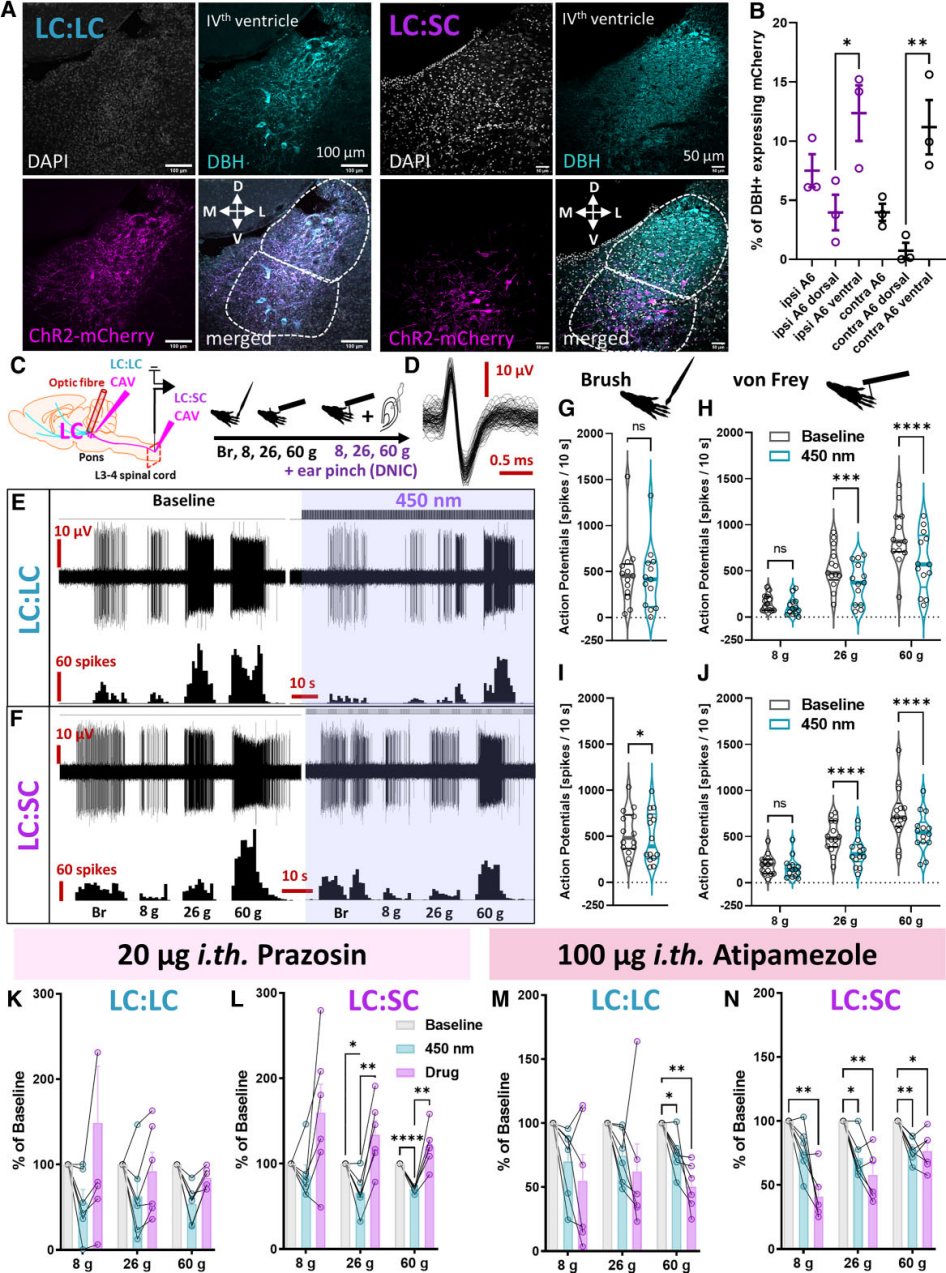
**A ventral cerulean neuronal population inhibits spinal nocioceptive processing via α_1_-ARs.** (**A**) Immunohistochemical analysis of LC dopamine-β-hydroxylase (DBH)-expressing noradrenergic neurons transduced by CAV delivering channelrhodopsin-2-mCherry construct under catecholamine specific promoter (PRS) injected locally (LC:LC module) or in the ipsilateral lumbar dorsal horns (LC:SC module). (**B**) Percentage of mCherry-expressing DBH neurons in the ipsi- and contralateral LC following LC:SC module labelling. Mean ± SEM of *N* = 3 animals per group, *n* = 6–8 slices per animal, unpaired one-way ANOVA performed on *N*, (structure) *P* = 0.0018, *F*(5,12) = 7.747. (**C**) Schematic of the *in vivo* electrophysiological experiments. (**D**) WDR neuron units code upon stimulation with natural stimuli. (**E**) WDR neuron inhibition following LC:LC module ChR2-mediated activation (450 nm laser pulses: 5 Hz, 20 ms and 238 mW/mm^2^). (**F**) The equivalent LC:SC module activation is shown. Quantification of (**G**) brush and (**H**) von Frey evoked activity before/after LC:LC module activation. Brush and von Frey: mean ± SEM of *N* = 13 animals per group, *n* = 13 cells per group; paired *t*-test performed on *n*: *P* > 0.05 (brush) and two-way ANOVA (von Frey) performed on *n*, (von Frey) *P* < 0.0001, *F*(2,36) = 24.37, (450 nm) *P* < 0.0001, *F*(1,36) = 47.29. Quantification of (**I**) brush and (**J**) von Frey evoked activity before/after LC:SC module activation. Brush and von Frey: mean ± SEM of *N* = 14 animals per group, *n* = 14 cells per group; paired *t*-test performed on *n*, *P* < 0.05 (brush); two-way ANOVA (von Frey) performed on *n*, (von Frey) *P* < 0.0001, *F*(2,39) = 23.75, (450 nm) *P* < 0.0001, *F*(1,39) = 89.83. Prazosin (α_1_-AR antagonist) reversed the inhibitory effect of (**K**) LC:LC and **(L**) LC:SC module activation. LC:LC or LC:SC prazosin: mean ± SEM shown as percentage of baseline for *N* = 6 animals per group, one cell per animal; two-way ANOVA with Geisser-Greenhouse correction (LC:LC-group) *P* > 0.05, F(1.03,5.17) = 3.306 and (LC:SC-group) *P* < 0.01, *F*(1.23,6.17) = 14.24, respectively. (**M**) LC:LC- and (**N**) LC:SC-mediated inhibition of WDR neurons was further potentiated after spinal application of 100 µg atipamezole (an α_2_-AR antagonist). LC:LC or LC:SC atipamezole: mean ± SEM shown as percentage of baseline for *N* = 6 animals per group, one cell per animal; two-way ANOVA with Geisser-Greenhouse correction (LC:LC-group) *P* < 0.05, *F*(1.39,6.94) = 5.635, and (LC:SC-group) *P* < 0.001, *F*(1.82,9.09) = 26.58. Tukey *post hoc* test used for all ANOVAs: *P < 0.05, **P < 0.01, ***P < 0.001, ****P < 0.0001. See Supplementary Fig. 1 and Supplementary Table 1.

Hypothesizing that the contrasting impact of spinal atipamezole on spinal neuronal activity following activation of a descending noradrenergic control^4,5^ reflected the activation of discrete rather than identical top-down modulatory circuits, we microinjected CAV spinally (thus retrogradely labelling an LC:SC module) or in the LC itself (thus labelling an LC:LC module) to deliver ChR2 under a noradrenergic promoter^12^ (Fig. 1A). After confirming ipsilateral ventral LC labelling following spinal CAV injection (12.4 ± 2.4% ventral versus 4.0 ± 1.5% dorsal noradrenergic LC neurons expressed mCherry; Fig. 1B and Supplementary Table 2) as performed in the previous study,^4,6^ we demonstrated that optoactivation of both LC:LC and LC:SC modules inhibited mechanically-evoked spinal WDR neuron activity (Fig. 1C and Supplementary Fig. 1), while stimulus intensity coding (8, 26 and 60 g von Frey) was maintained (Fig. 1D–J). LC-mediated inhibition of WDR neuronal activity upon stimulation with mechanical modalities was reversed by the α_1_-AR antagonist prazosin (Fig. 1K and L) but enhanced by local application of atipamezole (Fig. 1M and N). Our results suggest that phasic activation of the LC inhibits spinal WDR neuron activity via an α_1_-AR-mediated mechanism. Unlike α_2_-ARs, which directly mediate inhibition by coupling with small Gi proteins, α_1_-ARs couple with facilitatory G-proteins.^10^ Therefore, α_1_-AR-mediated inhibition of WDR neurons is likely to be indirect, for example via noradrenergic activation of inhibitory interneurons therein. In fact, activation of α_1_-ARs expressed on spinal GABAergic interneurons has been reported previously as a plausible mechanism for descending coerulean inhibitory controls.^11,13^

### DNIC expression is inhibited by LC:SC module optoactivation

**Figure 2:**
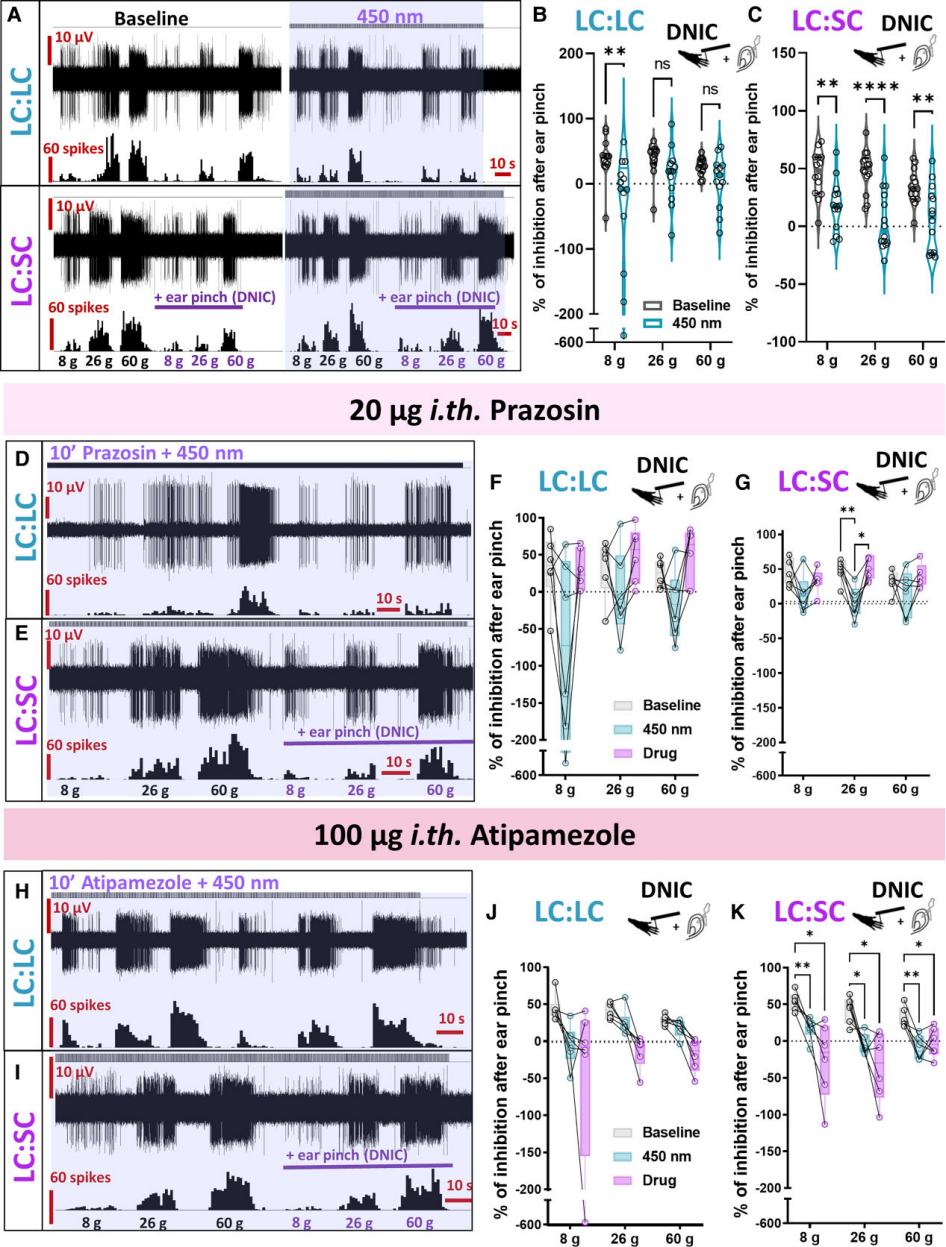
**DNIC expression is inhibited by LC:SC module optoactivation.** (**A**) DNIC expression, quantified as the inhibitory effect of a conditioning stimulus (ear pinch), decreased following LC:LC module optoactivation (450 nm laser pulses), and was abolished following identical LC:SC module activation. (**B**) Percentage of inhibition after DNIC activation before/after LC:LC module activation. Mean ± SEM of *n* = 13 animals per group, *n* = 13 cells per group; two-way ANOVA performed on *n*, (450 nm) *P* < 0.01, *F*(1,36) = 10.75. (**C**) Identical experiments before/after the LC:SC module activation. Mean ± SEM of *n* = 14 animals per group, *n* = 14 cells per group; two-way ANOVA performed on *n*, (450 nm) *P* < 0.0001, *F*(1,39) = 46.01. Prazosin partially reversed the impact of (**D**) LC:LC or (**E**) LC:SC module activation on DNIC expression: (**F**) LC:LC prazosin: mean ± SEM shown as percentage of baseline for *N* = 6 animals per group, one cell per animal; two-way ANOVA with Geisser-Greenhouse correction (group) *P* < 0.05, *F*(1.38,6.91) = 8.056. **(G**) LC:SC prazosin: mean ± SEM shown as percentage of baseline for *N* = 6 animals per group, one cell per animal; two-way ANOVA with Geisser-Greenhouse correction (group) *P* < 0.05, *F*(1.17,5.87) = 8.215. The inhibitory effect of (**H**) LC:LC and **(I**) LC:SC module activation on DNIC expression was facilitated by spinal atipamezole: (**J**) LC:LC atipamezole: mean ± SEM shown as percentage of baseline for *N* = 6 animals per group, one cell per animal; two-way ANOVA with Geisser-Greenhouse correction (group) *P* > 0.05, *F*(1.06,5.31) = 4.950. **(K**) LC:SC atipamezole: mean ± SEM shown as percentage of baseline for *N* = 6 animals per group, one cell per animal; two-way ANOVA with Geisser-Greenhouse correction (group) *P* < 0.001, *F*(1.82,9.01) = 26.58. Tukey *post hoc* test used for all ANOVAs: *P < 0.05, **P < 0.01, ****P < 0.0001. See Supplementary Table 1.

Hypothesizing that the impact of atipamezole on WDR activity in the study by Hirschberg and colleagues^4^ was a result of the malfunction of another non-coerulean inhibitory noradrenergic control, we investigated the impact of LC:LC or LC:SC module optoactivation on the expression of DNIC, where DNICs were previously shown to be abolished by spinal atipamezole.^3^ Interestingly, LC:SC optoactivation abolished DNIC, while LC:LC optoactivation only marginally decreased its potency (Fig. 2A–C). Meanwhile, while prazosin partially restored the LC:LC or LC:SC optoactivation-evoked decrease in DNIC expression (Fig. 2D–G), atipamezole facilitated it (Fig. 2H–K). We propose that, upon optoactivation of the LC:LC module, it is likely that a proportion of dorsal and ventral LC neurons are stimulated, causing a decrease in DNIC potency due to communication between the LC and the DNIC origin nucleus. Thus, it is likely that the dorsal LC has either no effect or facilitates DNIC functionality, while the LC:SC direct pathway inhibits WDR activity via spinal α_1_-ARs with simultaneous direct brainstem-located inhibition of the DNIC origin nucleus.

### Ablation of coerulean noradrenergic fibres does not affect basal spinal convergent neuron activity nor DNIC expression

**Figure 3:**
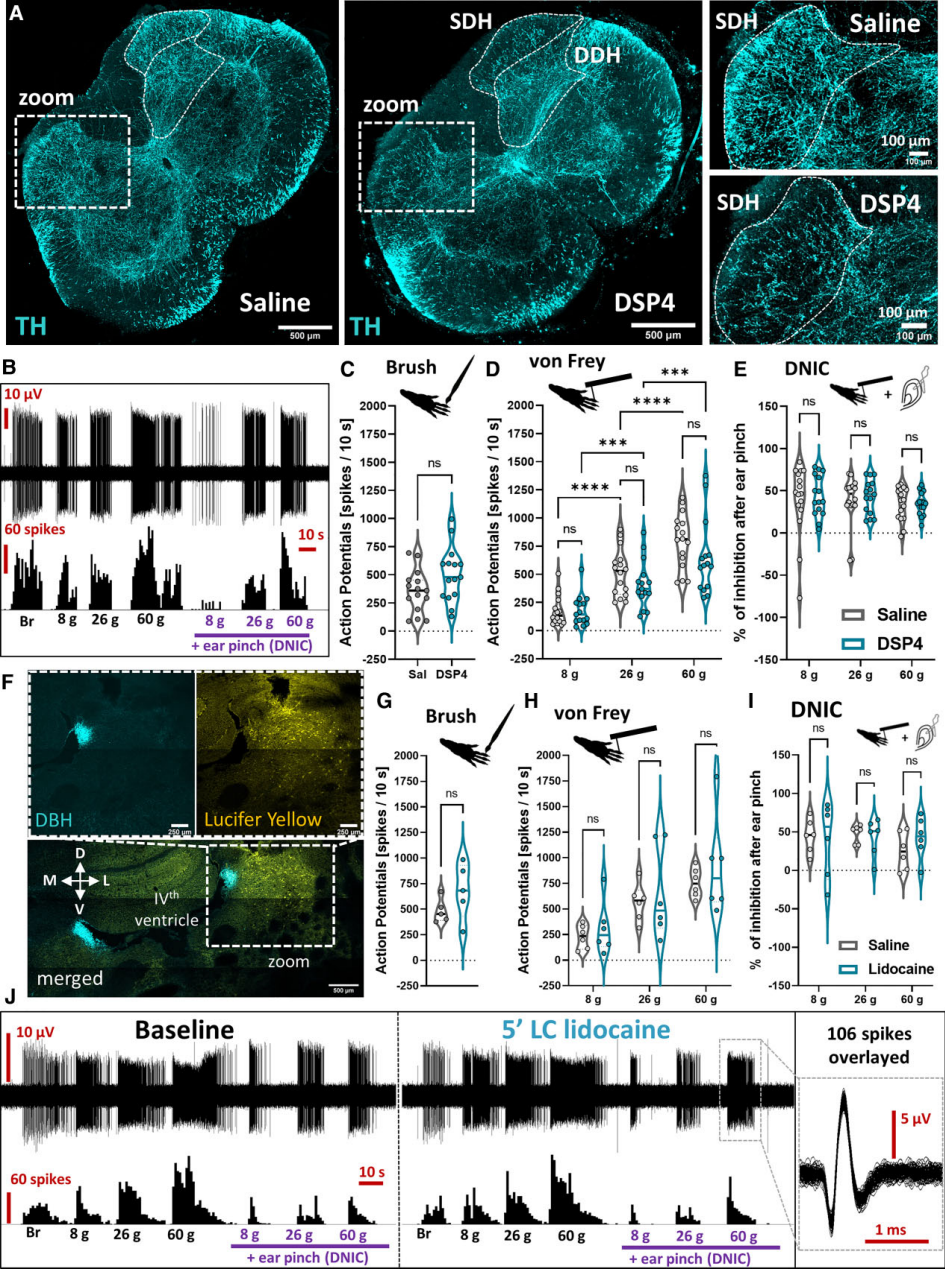
**Ablation of coerulean noradrenergic fibres does not affect basal spinal convergent neuron activity or DNIC expression.** (**A**) A PACT-cleared 500 µm thick lumbar spinal cord section (saline versus DSP4-treated rats) evidences a decrease in tyrosine hydroxylase (TH) immunolabelled fibres in the superficial but not deep dorsal horn (SDH/DDH). (**B**) DSP4 treatment did not impact WDR neuron sensory coding nor DNIC expression. Quantification of (**C**) brush and (**D**) von Frey evoked action potentials in saline and DSP4 treated rats. Brush and von Frey: mean ± SEM of *N* = 6 animals per group, *n* = 15 cells per group; unpaired *t*-test performed on *n*: *P* > 0.05 (brush); two-way ANOVA (von Frey) performed on *n*, (von Frey) *P* < 0.0001, *F*(2,30) = 128.7, (DSP4) *P* > 0.05, *F*(1,15) = 1.851. **(E**) Percentage of inhibition after DNIC activation as shown in (**B**). DNIC: mean ± SEM of *N* = 6 animals per group, *n* = 15 cells per group; two-way ANOVA performed on *n*, (DSP4) *P* > 0.05, *F*(1,15) = 0.2105. (**F**) Analogously to DSP4 treatment, ipsilateral LC microinjection of 2% lidocaine (marked by lucifer yellow) does not affect WDR neuronal activity nor DNIC expression, as quantified in (**G**) for brush and (**H**) for von Frey. Brush: mean ± SEM of *N* = 5 animals per group, *n* = 5 cells per group; paired *t*-test performed on *n*: *P* > 0.05. von Frey: mean ± SEM of *N* = 6 animals per group, *n* = 6 cells per group; two-way ANOVA performed on *n*, (von Frey) *P* < 0.001, *F*(2,10) = 23.97, (DSP4) *P* > 0.05, *F*(1,5) = 0.78. (**I**) Percentage of inhibition after DNIC activation as shown in **(J**). DNIC: mean ± SEM of *N* = 6 animals per group, *n* = 6 cells per group; two-way ANOVA performed on *n*, [DSP4] *P* > 0.05, *F*(1,5) = 0.063. Tukey *post hoc* test used for all ANOVAs: *P < 0.05, **P < 0.01, ****P < 0.0001. See Supplementary Table 1.

To further investigate this separation of LC:SC and DNIC pathway functionality, we systemically injected the neurotoxin DSP4 to deplete noradrenergic projections from the LC^16,17^ (Fig. 3A). No impact on WDR activity was observed 18–20 days following treatment (Fig. 3B–D), and DNICs were expressed (Fig. 3E). Elsewhere, we microinjected lidocaine (a sodium channel blocker) to the LC ipsilateral to the recorded WDR neuron (Fig. 3F). This had no effect on evoked WDR activity (Fig. 3G and H) or on DNIC expression (Fig. 3I and J). These results suggest that tonic activity in the LC (i) is not required to maintain DNIC expression; and (ii) does not modulate basal stimulus-evoked firing of spinal WDR neurons in health.

## Discussion

Herein we evidence that in health, phasic activity of the LC (upon LC:LC or LC:SC optoactivation) inhibits spinal WDR neurons via an α_1_-AR-mediated mechanism, while discrete LC:SC optoactivation abolishes the expression of DNIC, a brainstem to spinal cord pathway that inhibits spinal WDR neurons via an α_2_-AR-mediated mechanism. Tonic LC activity has no effect on DNIC expression or on the tonic modulation of the WDR neuronal firing rate.

Our study points towards an interaction but functional distinction between DNIC and LC-spinal cord pathways. Both are encompassed by the descending pain modulatory system whose output likely represents a conglomerate of operationally unique systems engaged by discrete circuits that are each influenced differentially by sensory drivers. If true, this would have consequences for the way in which targeted pain management is prescribed in chronicity, where we know that descending pain modulatory system-restorative pharmacotherapies do not alleviate pain in all patients. This ‘one size does not fit all’ phenomenon is unsurprising given the complexity of the circuits therein. For example, the coerulean neuronal population is developmentally diverse,^18^ and distinct anatomical projections from within^5^ mediate discrete aspects of the sensory and affective experience.^19,20^ The LC’s modular functional organization^4–6^ thus lends itself to facilitatory as well as inhibitory influences on spinal nocioceptive activity, where the underlying mechanism(s) involved will include neuro-immune interactions, since superficial dorsal horn astrocytes expressing α_1_-ARs were shown to be critical analgesic regulators in monoaminergic transmission terms.^21^

In the present study, our demonstration that activating the LC:SC pathway in health abolishes DNIC, while LC neuronal ablation does not point towards the likelihood of maladaptive communication between LC and DNIC circuits being an underlying mechanism of certain chronic pain phenotypes. DNICs are not expressed in varied animal models of chronic pain,^3,22,23^ and we have demonstrated previously that DNIC is expressed in a disease-stage specific manner in rodent models of osteoarthritis and cancer-induced bone pain.^22,23^ Disease-related changes to descending modulatory controls likely impact endogenous inhibitory modulation in the long term. If it was evidenced that a noradrenergic drive from the LC exacerbates pain in early stages of disease, one could envisage that the therapeutic application of pharmacological manipulators of specific spinal adrenoceptors would benefit patients at certain stages of specific diseases (and we include a review of the spinal anatomical distribution of ARs, including consideration of single cell RNAseq data tied to a prediction of the potential mechanisms involved, in Supplementary Fig. 2). Indeed, previous research has demonstrated abolished DNIC expression in the late stage of a model of chronic joint inflammatory pain and impaired descending noradrenergic modulation with relation to the LC.^24^ This insight, specifically linking stage-specific DNIC attenuation to impaired LC functionality, lends weight to the argument that communication between LC and DNIC origin nuclei governs the final output of descending modulatory controls that are subserved by noradrenaline. However, the nature of the influence is unknown, and a future research goal includes employing genetic strategies to determine the nature of the neuronal populations that mediate crosstalk between the LC and DNIC origin nuclei.

Summarizing, defining the functional relationship between the LC and DNIC-origin nuclei will allow identification of the underlying circuitry responsible for descending inhibitory controls in health and their perturbation in chronic pain. Do chronic pain inducing diseases lead to altered brainstem nucleus crosstalk and/or spinal pharmacological functionality that is specific in terms of disease type and stage? Revealing novel mechanisms that underlie abnormal nocioceptive processing is the key to uncovering analgesic targets. Relevant to this, the work presented herein has uncovered a mechanism by which the body inhibits pain in an endogenous manner in health. Future studies will endeavour to uncover aspects of the postulated mechanism, whereby inferences regarding AR subtype involvement will require full elucidation of their anatomical distribution. The potential clinical relevance is apparent when considering that dysregulation of DNIC in rodent pain models translates to the clinic. Conditioned pain modulation, the proposed human counterpart of DNIC, is dysfunctional in chronic pain patients,^25,26^ and its maladaptive expression is associated with chronicity;^27^ translatable mechanisms between DNIC and conditioned pain modulation have been evidenced.^28^

Ultimately, the results of the present study may help to elucidate the origins of chronic pain where the LC, a complex multi-functional nucleus with a yet-to-be-fully-defined role in pain (especially chronicity), demands further investigation.

## Supplementary Material

awac085_Supplementary_Data

## Abbreviations

AR: adrenoceptor
CAV: canine adenovirus
ChR2: channelrhodopsin-2
DNIC: diffuse noxious inhibitory control
DSP4: *N*-(2-chloroethyl)-*N*-ethyl-2-bromobenzylamine hydrochloride
LC: locus coeruleus
LC:LC: coerulean module
LC:SC: coeruleospinal module
WDR: wide dynamic range
