# Supplementary material for: Distinct brainstem to spinal cord noradrenergic pathways inversely regulate spinal neuronal activity
Source: Brain. 2022 Mar 4;145(7):2293–300. doi: 10.1093/brain/awac085 (PMC9337805; doi:10.1093/brain/awac085)
Supplement: awac085_Supplementary_Data [file awac085_supplementary_data.pdf]

KING'S College LONDON

University of London

SUPPLEMENTARY MATERIAL

Wolfson CARD

Central Modulation of Pain
The Wolfson Wing
Hodgkin Building
Guys Campus
London, SE1 1UL

DISTINCT BRAINSTEM TO SPINAL CORD NORADRENERGIC PATHWAYS INVERSELY REGULATE SPINAL NEURONAL ACTIVITY

MATEUSZ W. KUCHARCZYK, FRANCESCA DI DOMENICO, AND KIRSTY BANNISTER

Supplementary Figures and Tables

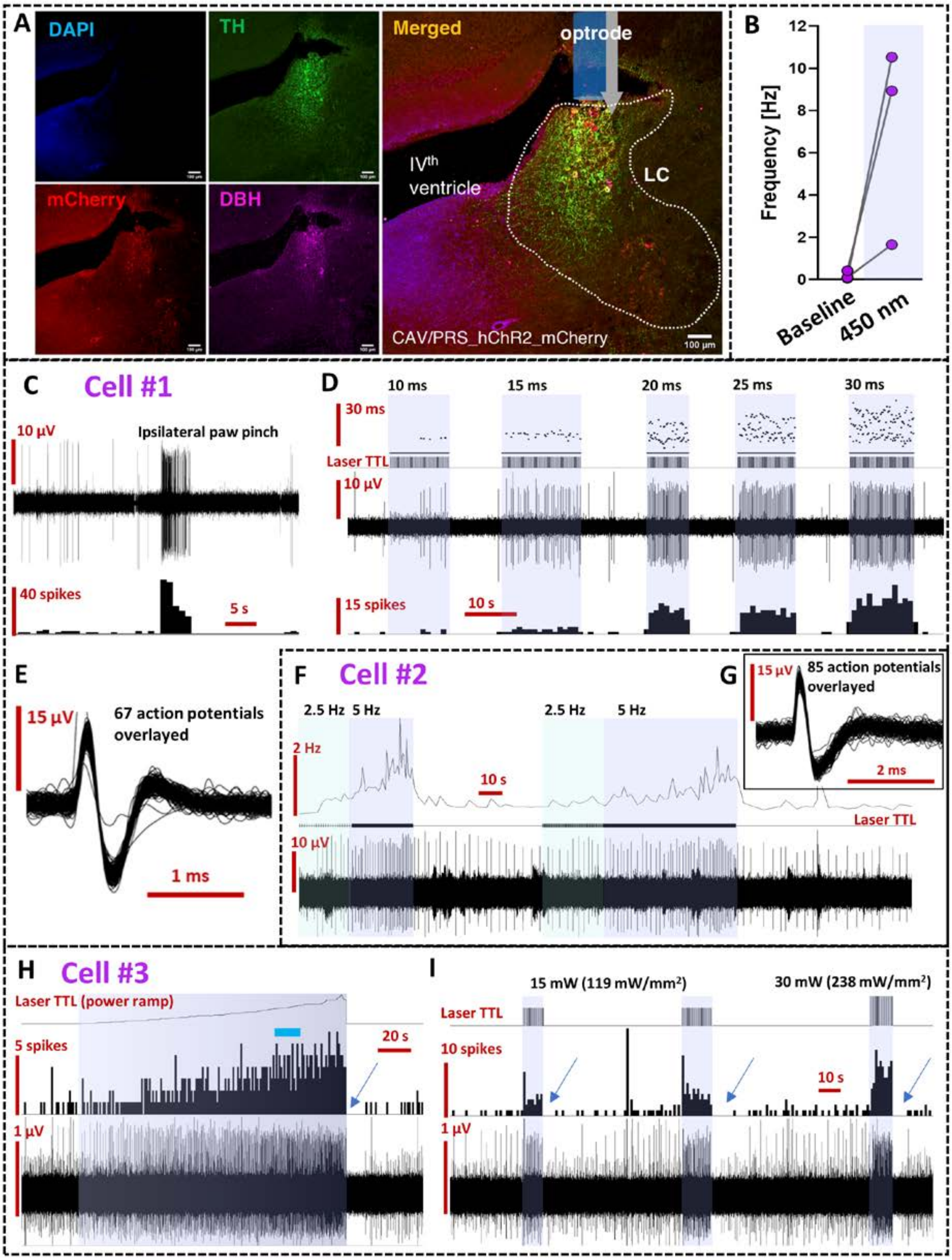

Supplementary figure 1. Optimisation of light parameters for locus coeruleus (LC) neuron optoactivation. A) A representative coronal section of transduced LC after in situ

injection of CAV/PRS-hChR2-mCherry. Tyrosine hydroxylase (TH) and dopamine-β-hydroxylase (DBH) immunolabelled cells overlapped with the virally-delivered mCherry in the LC proper. **B)** Mean firing frequency of transduced LC before and after optoactivation with chosen optimal 450 nm laser light parameters: 30 mW (238 mW/mm^2), 20 ms pulse, at 5 Hz. **C)** LC neuron responding to ipsilateral noxious pinch. Basal spontaneous discharges are shown before and after pinch. Note around 15 s inactivity period immediately after pinch. **D)** Same neuron as in b) optoactivated with different light pulse duration, but same frequency (5 Hz) and power (238 mW/mm^2). 20 ms pulse width was chosen as optimal. **E)** Overlayed action potentials of cell shown in c) and d). **F)** Optoactivation of the LC cell from different animal. Matching power (238 mW/mm^2) and pulse width (20 ms) was used, but with two different frequencies (2.5 and 5 Hz). 5 Hz was chosen optimal. **G)** An inclusion showing overlayed action potentials of the cell recorded in f). **H)** Light power ramp performed on another optically-sensitive LC neuron. An increase of discharges with the increase of laser power is shown. Blue bar indicates chosen power (238 mW/mm^2). Note a 15 s inactivity period immediately after laser light was turned off (blue arrow). **I)** Same cell as in h) showing light evoked responses with two different laser powers, but similar temporal pattern of pulses (20 ms width at 5 Hz).

Supplementary Table 1. Detailed statistics for Figure 1 – 3.

**Figure 1**		
**b)**	Mean±SEM of N=3 animals per group, n=6-8 slices per animal, unpaired One-Way ANOVA performed on N, [structure] $P<0.01$, $F_{(5, 12)}=7.747$.	Tuckey *post-hoc* used for all ANOVA: *P<0.05, **P<0.01, ***P<0.001, ****P<0.0001.
**g)**	Mean±SEM of N=13 animals per group, n=13 cells per group; Paired t-test performed on n: $P>0.05$.	
**h)**	Mean±SEM of N=13 animals per group, n=13 cells per group; Two-Way RM-ANOVA (vF) performed on n, [vF] $P<0.0001$, $F_{(2, 36)}=24.37$, [450 nm] $P<0.0001$, $F_{(1, 36)}=47.29$.	
**i)**	Mean±SEM of N=14 animals per group, n=14 cells per group; Paired t-test performed on n: $P<0.05$.	
**j)**	Mean±SEM of N=14 animals per group, n=14 cells per group; Two-Way RM-ANOVA (vF) performed on n, [vF] $P<0.0001$, $F_{(2, 39)}=23.75$, [450 nm] $P<0.0001$, $F_{(1, 39)} = 89.83$.	
**k)**	Mean±SEM shown as percentage of baseline for N=6 animals per group, one cell per animal; Two-Way RM-ANOVA with Geisser-Greenhouse correction: $P>0.05$, $F_{(1.03, 5.17)}=3.306$.	
**l)**	Mean±SEM shown as percentage of baseline for N=6 animals per group, one cell per animal; Two-Way RM-ANOVA with Geisser-Greenhouse correction: $P<0.01$, $F_{(1.23, 6.17)}=14.24$.	
**m)**	Mean±SEM shown as percentage of baseline for N=6 animals per group, one cell per animal; Two-Way RM-ANOVA with Geisser-Greenhouse correction: $P<0.05$, $F_{(1.39, 6.94)}=5.635$.	
**n)**	Mean±SEM shown as percentage of baseline for N=6 animals per group, one cell per animal; Two-Way RM-ANOVA with Geisser-Greenhouse correction: $P<0.001$, $F_{(1.82, 9.09)}=26.58$.	
**Figure 2**		
**b)**	Mean±SEM of N=13 animals per group, n=13 cells per group; Two-Way RM-ANOVA performed on n, [450 nm] $P<0.01$, $F_{(1, 36)}=10.75$.	Tuckey post-hoc used for all ANOVA: *P<0.05, **P<0.01, ****P<0.0001.
**c)**	Mean±SEM of N=14 animals per group, n=14 cells per group; Two-Way RM-ANOVA performed on n, [450 nm] $P<0.0001$, $F_{(1, 39)}=46.01$	
**f)**	Mean±SEM shown as percentage of baseline for N=6 animals per group, one cell per animal; Two-Way RM-ANOVA with Geisser-Greenhouse correction [group] $P<0.05$, $F_{(1.38, 6.91)}=8.056$	
**g)**	Mean±SEM shown as percentage of baseline for N=6 animals per group, one cell per animal; Two-Way RM-ANOVA with Geisser-Greenhouse correction [group] $P<0.05$, $F_{(1.17, 5.87)}=8.215$.	
**j)**	Mean±SEM shown as percentage of baseline for N=6 animals per group, one cell per animal; Two-Way RM-ANOVA with Geisser-Greenhouse correction [group] $P>0.05$, $F_{(1.06, 5.31)}=4.950$.	
**k)**	Mean ± SEM shown as percentage of baseline for N=6 animals per group, one cell per animal; Two-Way RM-ANOVA with Geisser-Greenhouse correction [group] $P<0.001$, $F_{(1.82, 9.01)}=26.58$.	
**Figure 3**		
**c)**	Mean±SEM of N=6 animals per group, n=15 cells per group: unpaired t-test performed on n: $P>0.05$.	Tuckey post-hoc used for all ANOVA: *P<0.05, **P<0.01, ****P<0.0001.
**d)**	Mean±SEM of N=6 animals per group, n=15 cells per group: Two-Way RM-ANOVA (vF) performed on n, [vF] $P<0.0001$, $F_{(2, 30)}=128.7$, [DSP4] $P>0.05$, $F_{(1, 15)}=1.851$.	
**e)**	Mean±SEM of N=6 animals per group, n=15 cells per group; Two-Way RM-ANOVA performed on n, [DSP4] $P>0.05$, $F_{(1, 15)}=0.2105$.	
**g)**	Mean±SEM of N=5 animals per group, n=5 cells per group; paired t-test performed on n: $P>0.05$.	
**h)**	Mean±SEM of N=6 animals per group, n=6 cells per group; Two-Way RM-ANOVA performed on n, [vF] $P<0.001$, $F_{(2, 10)}=23.97$, [DSP4] $P>0.05$, $F_{(1, 5)}=0.78$.	
**i)**	Mean±SEM of N=6 animals per group, n=6 cells per group; Two-Way RM-ANOVA performed on n, [DSP4] $P>0.05$, $F_{(1, 5)}= 0.063$.	

A

		Adra1a	Adra1b	Adra1d	Adra2a	Adra2b	Adra2c	Adrb1	Adrb2	Adrb3	Slc32a1	Slc17a6
	Glut1	0	11	0	1	1	0	2	0	0	3	186
CCK	Glut2	0	1	0	2	0	2	2	1	1	4	168
	Glut3	0	0	1	8	0	4	1	0	0	3	238
NTS	Glut4	0	1	0	1	0	2	1	0	3	2	171
	Glut5	0	0	0	3	0	1	3	0	3	0	183
NKB	Glut6	1	2	0	4	0	3	4	1	1	5	578
	Glut7	0	6	0	15	0	2	1	0	2	1	707
GRP	Glut8	0	0	0	6	0	4	2	0	0	1	376
	Glut9	0	2	0	8	1	1	4	0	1	0	255
SP	Glut10	0	2	0	133	7	8	7	0	0	3	683
	Glut11	3	1	0	73	0	6	3	0	3	6	457
	Glut12	0	2	0	8	1	5	2	0	0	0	179
	Glut13	0	0	0	4	0	3	4	0	0	6	389
	Glut14	3	6	0	7	0	13	3	0	2	4	350
	Glut15	0	0	0	1	0	3	2	0	0	8	144
Gal	Gaba1	0	0	0	1	0	0	0	0	0	12	12
Dyn	Gaba2	4	24	0	7	4	8	9	0	1	174	12
	Gaba3	13	11	0	9	13	24	4	0	0	243	16
	Gaba4	2	5	0	1	0	1	0	0	0	40	2
	Gaba5	3	3	0	1	0	1	1	0	0	43	0
NPY	Gaba6	4	1	0	8	1	5	1	0	0	107	1
	Gaba7	6	2	0	0	1	5	4	0	0	64	0
	Gaba8	6	2	0	2	20	8	3	0	0	111	1
Calret	Gaba9	1	7	0	5	9	11	12	0	0	143	4
	Gaba10	1	3	1	3	0	2	1	0	0	107	1
	Gaba11	3	4	1	11	0	5	0	0	0	113	20
	Gaba12	1	13	0	2	2	9	2	0	0	123	1
	Gaba13	3	7	0	2	1	3	1	0	1	93	1
Parvalb	Gaba14	0	0	1	13	2	1	2	0	0	85	11
	Gaba15	1	2	0	4	0	1	1	0	0	28	0

B

(all)	GLUT	GABA
Adra1a	7	48
Adra1b	34	84
Adra1d	1	3
Adra2a	274	69
Adra2b	10	53
Adra2c	57	84
Adrb1	41	41
Adrb2	2	0
Adrb3	16	2

Slc32a1	46	1459
Slc17a6	5064	83

C

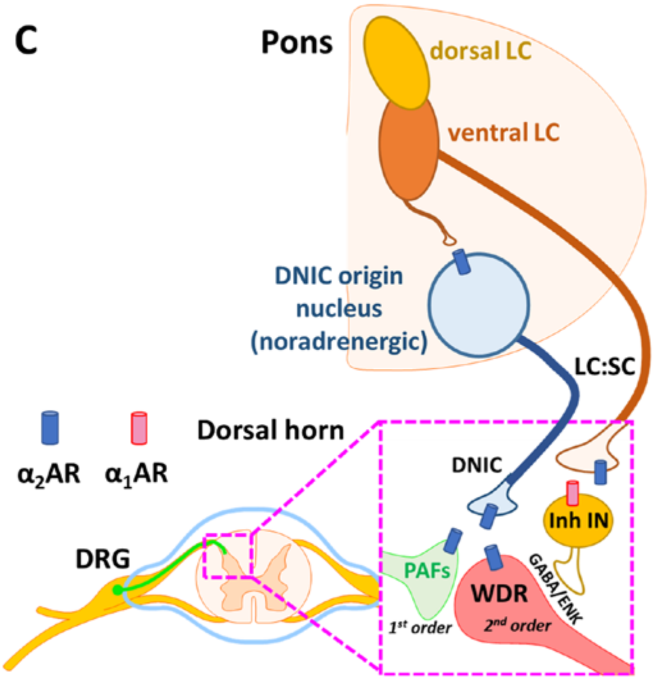

Supplementary figure 2. Suggested mechanism for DNIC – LC:SC pathways interaction. **A)** Genes analysed in the spinal cord single cell RNAseq[1]. All cells with 0 counts for *Gapdh* were excluded from analysis (total 25 from 1545 cells: 18 out of 767 Glut, 7 out of 778 GABA, were excluded). Intensity is colour coded within a row. Based on scRNAseq data a low level

of RNA copies was detected for *Adra1b*, *Adra2a*, and *Adra2c* on spinal neurons, with a biased expression for excitatory *Adra1b* towards GABAergic inhibitory spinal neurons, and inhibitory *Adra2a* expression was biased towards glutamatergic excitatory spinal neurons. This suggests a net inhibitory action for spinal noradrenaline either *via* direct inhibition of excitatory neurons (*via* α_2-ARs) or indirectly by the facilitation of the inhibitory interneurons therein (*via* α_1-ARs). *In situ* hybridisation data from the Allen Brain Atlas supports this expression pattern. Additionally, adrenoceptors are present on descending fibre terminals where they function as autoreceptors (see datasets in Allen Brain Atlas, www.proteinatlas.org, and review[2]), as well as on primary afferents (mouse DRG neurons express only one type of adrenoceptor: *Adra2c*[3]). Furthermore, a significant expression of *Adra1d* is reported in the Allen Brain Atlas's *in situ* hybridisation data (cell type unspecific, thus likely non-neuronal), while others' RNAseq data[4] suggests a significant expression of *Adra1a* on *Hes1*+ astrocytes in the dorsal lumbar dorsal horn, which role was tied to coerulean control of mechanical hypersensitivity. **B)** Summed counts from all subtypes of Glut and GABA neurons shown in a). **C)** Schematic representation of the postulated mechanism. The LC:SC pathway originates in the ventral LC, and this pathway inhibits WDR neurons indirectly *via* α_1-AR mediated activation of spinal inhibitory interneurons. Atipamezole facilitates this inhibition (but only upon phasic LC:SC activation), possibly by blocking autoreceptors on descending fibre terminals potentiating further release of noradrenaline. Prazosin blocks LC:SC mediated inhibition. Additionally, phasic activation of the LC:SC pathway inhibits the DNIC origin nucleus, presumably *via* α_2-ARs therein. Activation of DNIC (also phasic) in naïve animals inhibits WDR neurons by α_2-ARs-mediated mechanism. These receptors may be located either directly on the WDR neurons or may have an indirect impact (for example via presynaptic inhibition of sensory afferents). Note the body of evidence regarding protein distribution of adrenoceptors in the cord[5–8], which is consistent with the RNAseq data (**A, B** above) despite known low specificity (i.e. knock-out unvalidated) of adrenoceptors antisera[9]. From this we infer that the majority of α_2-ARs immunoreactivity in the cord has peripheral sensory neuron origin, as confirmed by the significant signal reduction in animals with ablated sensory neurons (either by dorsal rhizotomy or perinatal capsaicin treatment)[8]. **Abbreviations: markers of glutamatergic neurons:** CCK-Cholecystokinin, NTS-Neurotensin, NKB-Neurokinin B, GRP-Gastrin-releasing peptide, SP-substance P, Slc17a6-Vesicular glutamate transporter 2 (VGluT2). **Markers for GABAergic neurons:** Gal-Galanin, Dyn-Dynorphin, NPY-Neuropeptide Y, Calret-Calretinin, Parvalb-Parvalbumin, ENK-enkephalin, Slc32a1-Vesicular inhibitory amino acid transporter (VGAT).

Supplementary Table 2. Comparison with labeling efficiency achieved in this and other papers following LC:SC module labelling.

LC:SC module (lumbar SC injections)	Howorth et al., 2009	Howorth et al., 2009	Howorth et al., 2009	Li et al., 2016	Li et al., 2016	Hirschberg et al. 2017	Kucharczyk et al., 2021 (this paper)	
	Fluorogold	holera toxin	.VV (hAdV-C5	CAV2	CAV2	CAV2	CAV2	
Titer used:	5%	1%	3x10e10 TU/mL	0.9x10e10 TU/ml	1.2x10e12 TU/ml	0.6x10e12 TU/ml	3x10e10 TU/ml	**Total number of NA neurons in the given structure (rat):**
Injected volume:	A pair of bilateral (50 nL each side) injections.	A pair of bilateral (500 nL each) injections.	2 pairs of bilateral injections, 500 nl each	2 pairs of bilateral injections, 500 nl each	2 pairs of bilateral injections, 500 nl each	6 injections of 400 nl unilaterally	3 injections, 400 nl each, unilaterally	
A5 ipsi	11.90%	4.40%	1.20%	2%	6.60%	N/A	N/A	**1552 ± 337** (Howorth et al., 2009)
A6 ipsi	7.50%	4.90%	3.50%	11%	16.70%	11.60%	16%	**3268 ±114** (Loughlin et al., 1986)
A6 dorsal	N/A	N/A	N/A	N/A	N/A	N/A	12.40%	
A6 ventral	N/A	N/A	N/A	N/A	N/A	N/A	4.00%	
A7 ipsi	17.60%	13.60%	3.20%	25%	24.60%	N/A	N/A	**346 ± 43** (Howorth et al., 2009)

Supplementary Materials and Methods

Animals

Male Sprague-Dawley rats (Envigo, UK) were used for experiments. Animals were group housed on a 12:12 h light–dark cycle. Food and water were available *ad libitum*. Animal house conditions were strictly controlled, maintaining stable levels of humidity (40–50%) and temperature (22 ± 2°C). All procedures described were approved by the Home Office and adhered to the Animals (Scientific Procedures) Act 1986. Every effort was made to reduce animal suffering and the number of animals used was in accordance with International Association for Study of Pain (IASP)[10] and ARRIVE ethical guidelines[11].

The observer was blind to DSP4 and LC:LC/LC:SC labelled experimental groups, but not to the pharmacological treatment performed during electrophysiological recordings. All surgical procedures were carried out under aseptic conditions and animals were maintained at 37˚C on a homeothermic pad. Brainstem tissue from injected animals was histologically analysed *post hoc* to confirm transduction of LC neurons (fluorescent tag expression).

All experiments were designed to contain minimum of 6 rats per group, based on G-power predictions from previous experiments. Animals were randomly assigned to experimental groups. From 60 rats designated for this study, 7 failed to provide stable WDR neuronal recordings, 3 rats developed vestibular problems reaching humane endpoint within 4 days after LC virus microinjection, and 1 animal died 24 hours after DSP4 administration. In total 49 rats were used as follows: 24 rats were used for mixed opto-pharmacology experiments (6 rats per group: LC:LC atipamezole, LC:LC prazosin, LC:SC atipamezole, LC:SC prazosin). Additional 3 rats were used for WDR baseline characterisation with optogenetics (2 for LC:SC, 1 for LC:LC) followed by LC optoelectrical recordings of transduced neurons. In the latter no pharmacology was performed. Further 6 rats were used for DSP4 group and 15 rats were used as naïve controls, which included 6 rats used for lidocaine microinjection experiment.

DSP4 injections

For ablation of the coerulean noradrenergic fibres across the neuroaxis, a selective neurotoxin N-(2-chloroethyl)-N-ethyl-2-bromobenzylamine (DSP4) hydrochloride (Sigma, Dorset, UK)

was used[12,13]. Six rats weighting 60-80 g were injected intraperitoneally with freshly prepared 50 mg/kg DSP4 in saline. Additional six control rats received vehicle injection. 15% lethality rate from the DSP4 toxin is expected[14], therefore, to minimise eventual animals suffering injections were given early on the day and rats were kept for up to 4 hours in the recovery incubator (set at 32°C) and carefully monitored for up 8 hours to minimise lethality. 14-16 days after the toxin injection terminal electrophysiological characterisation of lumbar deep dorsal horn WDR neurons was performed followed by transcardial perfusion with cold saline followed by 4% PFA and tissue collection for histological analysis.

Virus injections

The LC:LC module

To transduce catecholaminergic coerulean neurons, ipsilateral LC stereotaxic injections were made (Kopf Instruments, UK) analogously to described in detail earlier[15]. In brief, male Sprague-Dawley rats (180-220 g, Charles River) were anesthetized with a mix of ketamine (5 mg/100 g, Vetalar; Pharmacia) and medetomidine (30 µg/100 g, Dormitor; Pfizer) delivered intraperitoneally until loss of paw withdrawal reflex and perioperative analgesia was achieved by the subcutaneous injections of meloxicam (2 mg/kg, Metacam®, Boehringer Ingelheim, Berkshire, UK). The animal was placed in a stereotaxic frame and core temperature was maintained at 37°C using a homeothermic blanket (Harvard Apparatus, US). Aseptic surgical techniques were used throughout, and eyes were protected with sterile paraffin-based moisturiser (Lacri-Lube, Allergan, UK). Using a 0.7 mm dental drill a hole was made in the skull right above the targeted structure and dura was carefully pierced with the aid of surgical microscope until the visible amount of CSF appeared. With 10° rostral angulation to avoid puncturing the sinus, the following coordinates were used (from lambda): RC: -2.1 mm, ML: 1.3 mm, and -5.8-6.2 mm deep from the cerebellar surface. A glass pulled micropipette coupled to electronically controlled nanoinjector (Nanoliter 2010, WPI, FL, US) with back-filled mineral oil as a medium was used for precise delivery of canine adenovirus (CAV) carrying channelrhodopsin 2 under the control of catecholamine-specific synthetic promoter (sPRS) (CAV-sPRS-hChR2(H134R)-mCherry, titer >$3x10^{10}$ TU/ml, PVM, Montpellier, a gift from Professor Anthony Pickering, University of Bristol[15,16]). The micropipette maintaining 20-40 µm tip diameter was front loaded with the virus immediately prior the injection. Three

injections, each of 400 nl, were made every 200 μm starting from -6.2 mm (DV) with 2 nl/s delivery rate and minimal 3-5 minutes were allowed between slow pipette retraction. The wound was irrigated with saline and closed with Vicryl 4-0 absorbable sutures and wound glue (VetBond, 3M, UK). Anaesthesia was reversed with i.p. injection of atipamezole (Antisedan, 0.1 mg/100 g; Pfizer, UK). The animals were placed in a thermoregulated recovery box until fully awake. Two to three weeks were allowed for the transgene expression, after which animals were taken for terminal *in vivo* electrophysiology. Labelling efficiency of the LC:LC module was comparable to the previously reported[16–18].

The LC:SC module

To transduce spinally projecting catecholaminergic brainstem neurons, the same CAV-sPRS-hChR2(H134R)-mCherry virus (titer >3x10^{10} TU/ml,[16]) was injected in the lumbar spinal cord. In brief, male Sprague-Dawley rats weighting 60-80 g were used for injections. Following the induction of anaesthesia (5% isoflurane in 1 l/min oxygen) rats were placed in a stereotaxic frame (without fixing the head) and maintained with 1.8-2% isoflurane in oxygen (1 l/min flow) delivered via nose cone. Core temperature was maintained at 37°C using a homeothermic blanket (Harvard Apparatus, US), aseptic surgical techniques were used throughout and eyes were protected with sterile paraffin-based moisturiser (Lacri-Lube, Allergan, UK). The subcutaneous injections of meloxicam (2 mg/kg, Metacam, Boehringer Ingelheim, Berkshire, UK) were used for perioperative analgesia. After a complete loss of paw withdrawal reflex the 2-2.5 cm skin overlaying lumbar spinal cord was incised and overlaying muscles carefully dissected to gain access to T12/L1 intervertebral space. The sterile spinal clamp was used to secure the cord and the spinal L3-L4 region was exposed by bending the cord rostrally providing easy access to the underlaying dura without the need for extensive laminectomy. A small puncture in the dura was made with the aid of surgical microscope and a glass pulled micropipette (20-40 μm tip diameter) was inserted in the dorsal horn to carefully deliver CAV virus at 2 nl/s with the aid of electronically controlled nanoinjector (Nanoliter 2010, WPI, FL, US). Three injections, each of 400 nl, were made ipsilaterally at around 300-400 μm apart in the rostrocaudal axis, 200 μm lateral from the central vessel, and at 800, 600 and 400 μm deep from the cord surface for injection 1-3, respectively. After each injection minimum of 5 minutes were allowed for the virus diffusion in the cord parenchyma followed by slow pipette retraction. The surgical area was maintained moist with the aid of sterile saline. The wound was closed with sterile wound clips (removed at day 7 post-surgery) and surgical glue

(VetBond 3M, UK). The animals were placed in a thermoregulated recovery box until fully awake. Two to three weeks were allowed for the transgene expression, after which animals were taken for terminal *in vivo* electrophysiology.

Spinal cord *in vivo* electrophysiology

In vivo electrophysiology was performed on animals weighing 240–300 g as previously described[19]. Briefly, after the induction of anaesthesia, a tracheotomy was performed, and the rat was maintained with 1.5% of isoflurane in a gaseous mix of N_2O (66%) and O_2 (33%). Core body temperature was monitored and maintained at 37°C by a heating blanket unit with differential rectal probe system. Electrocardiogram (ECG) was monitored by two intradermal needles inserted in front limbs with signal amplified by the Neurolog system consisting of AC preamplifier (Neurolog NL104, gain x200), through filters (NL125, bandwidth 300 Hz to 5 KHz) and a second-stage amplifier (Neurolog NL106, variable gain 600 to 800) to an analogue-to-digital converter (Power 1401 625kHz, CED). Craniotomy was performed to gain stereotaxic access to the ipsilateral LC for either optic fibre or micropipette insertion as described in following sections. A laminectomy was performed to expose the L3–L5 segments of the spinal cord, the cord was clamped to minimise movement, dura was carefully removed with the aid of surgical microscope, and the recording area was secured by saline-filled well made in solidified 2% low melting point agarose (made in saline also). Using a parylene-coated, tungsten electrode (125 μm diameter, 2 MΩ impedance, A-M Systems, Sequim, WA, USA), wide dynamic range neurons in deep laminae IV/V (~650–900 μm from the dorsal surface of the cord) receiving intensity-coding afferent A-fibre and C-fibre input from the hind paw were sought by periodic light tapping of the glabrous surface of the hind paw. Extracellular recordings made from single neurones were visualized on an oscilloscope and discriminated on a spike amplitude and waveform basis. Specifically, the signal from the electrode's tip was processed via headstage connected to the neurolog system consisting of AC preamplifier (Neurolog NL104, gain x200), through HumBag (Quest Scientific, North Vancouver, Canada) used to remove low frequency noise (50–60 Hz), via a second-stage amplifier (Neurolog NL106, variable gain 600 to 800), filters (NL125, bandwidth 1000 Hz to 5 kHz) and spike-trigger (Neurolog NL106, variable gain 600 to 800) to an analogue-to-digital converter (Power 1401 625 kHz, CED). Spike trigger was visualised on a second oscilloscope channel and manually set to follow single unit spikes. Its analogue signal was digitalised via event input to build stimulus histogram in real time along the waveform recordings. All the data were

captured by an analogue-to-digital converter (Power 1401 625 kHz, CED) connected to a PC running Spike2 v8.02 software (Cambridge Electronic Design, Cambridge, UK)) for data acquisition, analysis and storage. Simultaneous ECG monitoring and transistor–transistor logic (TTL) triggers (i.e. for the lasers, see below) were additionally coupled to Spike 2 recording traces via CED-1401 analogue inputs.

Stimulation paradigm in all electrophysiological recordings

Natural mechanical stimuli, including brush and von Frey filaments (8 g, 26 g and 60 g) and von Frey filaments with concurrent ipsilateral noxious ear pinch (15.75 × 2.3 mm Bulldog Serrefine ear clip; InterFocus, Linton, United Kingdom), were applied in this order to the receptive field for 10 s per stimulus. The noxious ear pinch was used as a conditioning stimulus (CS) to trigger diffuse noxious inhibitory control (DNIC[20]) and was quantified as an inhibitory effect on neuronal firing during ear pinch to its immediate respective von Frey filament applied without the conditioning stimulus (% of inhibition after ear pinch). A minimum 30 s non-stimulation recovery period was allowed between each test in the trial. A 10-minute non-stimulation recovery period was allowed before the entire process was repeated for control trial number 2 and 3. The procedure was repeated 3 times and averaged only when all neurons met the inclusion criteria of 10% variation in action potential firing for all mechanically evoked neuronal responses. No animals were excluded from analysis.

In vivo spinal pharmacology with electrophysiological monitoring

After collection of predrug baseline control data as outlined above, atipamezole (a α_2-AR antagonist: 100 μg; Sigma-Aldrich, Gillingham, United Kingdom, dissolved in 97% normal saline, 2% Cremophor [Sigma, UK], 1% dimethyl sulfoxide [DMSO; Sigma, UK] vehicle), prazosin hydrochloride (α_1-AR antagonist: 20 μg, Sigma-Aldrich, Gillingham, United Kingdom, dissolved in water for injections) was administered topically to the spinal cord in 50 μl volumes following gentle removal of residing saline in the agarose well. Each individual drug dose effect (one stable neuron assessed per rat) was followed for up to 60 minutes with tests performed typically at 3 time points (starting at 10, 20 and 30 minutes). For each time point, a trial consisted of consecutive stable responses to brush, von Frey and DNIC (von Frey with concurrent ipsilateral ear pinch). The post-drug effects in subsequent individual modalities (as compared to mean pre-drug baseline) were judged by the maximal change in recorded

action potential rate. All data plotted represents the time point of peak change based on these criteria.

Atipamezole is a selective α_2-adrenoceptor antagonist (α_2/α_1 ratio is >8300)[21], while prazosin is a selective α_1-adrenoceptor antagonist (α_1/α_2 ratio is >100-1600 https://www.guidetopharmacology.org[22]. Doses for the antagonists were chosen based on previously published reports: where 100 μg of atipamezole was doubled the substantial dose needed for the reversal of antinociceptive action of intrathecal clonidine[15] or chemogenetic (PSAM-mediated) activation of all descending noradrenergic controls[17]. This dose was also confirmed by several other reports[23] including ours[20,24]. Intrathecal prazosin was used by others in doses ranging from 10-40 μg[25,26]. For instance, 50-100 nmole (19-38 μg) of intrathecal prazosin fully reversed intrathecal noradrenaline effects in hot plate and tail flick behaviour in rats[26], where prazosin was 10 times more effective in reversing noradrenaline effects than yohimbine. The intrathecal effects for both antagonists are rapid, with the onset of 5-10 minutes and lasting for up to 45 minutes for atipamezole and over an hour for prazosin.

Lidocaine block of the LC activity

Six naïve rats weighting 240-260 g were used for lidocaine block of neuronal activity in the LC with simultaneous terminal spinal WDR neuron electrophysiological recordings. In brief, a hole in the skull was drilled right prior the lumbar laminectomy for DDH WDR neuronal recordings as described above. Utilising the same parameters to reach the ipsilateral to the spinal recording side ventral LC (-6.0 mm from the cerebellum surface), injections of lidocaine (2% in saline, Cat. L5647 Sigma, UK) were made after collecting stable baseline recordings of DDH WDR neurons. A glass pulled pipette was used coupled to the electronically controlled nanoinjector (Nanoliter 2010, WPI, FL, US) to precisely deliver the drug (500 nl, 2 nl/s). After the drug delivery, pipette was left in place throughout the spinal recordings. Tests were performed at 5-, 10- and 15-minutes post lidocaine injection. At the end of the experiment the solution in the pipette was replaced with 0.5% Lucifer Yellow-CH dipotassium salt (#L0144, Sigma, UK) and the pipette was re-inserted in the same brain region and 500 nl of the Lucifer Yellow Solution was injected therein. 10 minutes were allowed for the dye diffusion after which the animal was sacrificed by the anaesthetic overdose and immediate transcardial perfusion followed by brain extraction for anatomical verification post-mortem.

Optogenetics

Light stimulation of coerulean neurons during spinal WDR recordings

The 450 nm laser (Doric Lenses, Quebec, Canada) was externally TTL-triggered by the neurolog system (NeuroLog system, Digitimer, UK) to deliver defined light pulses (20 ms pulse width at 5 Hz). In brief, the laser light was coupled to a multimode 200 um patch cord (0.39 NA, #M75L01, Thorlabs, UK) and via SMA to SMA mating sleeve (#ADASMA, Thorlabs, UK) to second ferrule-terminating multimode 200 µm patch cord (0.39 NA, #M77L01, Thorlabs, UK) directly interconnected to multimode stainless steel 20 mm long cannula (200 µm diameter, 0.39 NA, #CFM12L20, Thorlabs, UK). The laser controller current was set to 44-54 mA adjusted (using PM16-130 power meter, Thorlabs, UK) to correspond to 30 mW (238 mW/mm^2) light power density at the tip of the implantable 200 µm fibre cannula[15]. The power density was adjusted for each preparation. After desired power was achieved the cannula was slowly inserted in the ventral LC ipsilateral to the recorded spinal WDR neurons. The canula was lowered using precise hydraulic micromanipulator (Narishige, Japan) with 10° rostral angulation at the following coordinates (from lambda): RC: -2.1 mm, ML: 1.3 mm, and -6.0 mm deep from the cerebellar surface.

Spinal WDR neurons were characterised by three stable baseline responses followed by three optically modulated responses. For combined optogenetics and spinal pharmacology, after collecting three stable baseline and three stable optoactivation responses (averaged, if stable), a drug (either 100 µg atipamezole or 20 µg prazosin) was applied topically on the exposed spinal cord surface, right above the recording site. To assess simultaneous action of the drug and the LC optoactivation, light pulses were delivered 30 s before and throughout each series of tests (approximately 5 minutes per series) and minimally 5 minutes of the recovery time was allowed between the tests. Pharmacology was monitored every 10 minutes for 30-40 minutes (each test with optoactivation) and the 60 minutes time point was to test neuron returning to the baseline (no optoactivation). At the end of every experiment, animals were sacrificed by the overdose of isoflurane and transcardially perfused with cold saline followed by 4% paraformaldehyde for anatomical evaluation.

LC neuron recording and optoactivation

A simultaneous recording and optical stimulation of the transduced LC neurons were made using microoptrodes as described earlier with minor modifications[27]. LC neurons were identified as described before[15] by their large amplitude with duration of action potentials over 1 ms, spontaneous firing (0.5-7 Hz) and biphasic response following hindpaw pinch (activation followed by transient silent period, particularly strong for the contralateral hindpaw). For the LC recordings and optoactivation, the all-glass recording microptrode with 20 µm tip diameter consisted of the recording core filled with 3 M sodium acetate (resulting in 2-3 MΩ resistance) and the parallel gradient index (GRIN) optical core (a gift from Professor Yves De Koninck, Laval University, Canada) coupled to the optic fibre (multimodal, 200 µm core diameter, 0.39 NA, #M77L01, Thorlabs, UK) was used. The LC neurons were optoactivated by 450 nm laser (Doric Lenses, Quebec, Canada) light pulses as described above[15]. With constant laser power set as 30 mW (238 mW/mm^2) and 5 Hz frequency we tested 10, 15, 20, 25 and 30 ms wide pulses, choosing 20 ms as the optimal pulse based on its almost maximal activation.

Immunohistochemistry

Animals were sacrificed by the overdose of anaesthetic and transcardially perfused with cold phosphate buffer saline (PBS) followed by 4% paraformaldehyde (PFA) in phosphate buffer (pH 7.5). Next, collected spinal cords and brains were post-fixed in 4% PFA for 3-4 days at 4°C, followed by 3-4 days at 4°C in 30% sucrose. Once tissue density equilibrated, lumbar spinal cords and brains were precut into 5 mm thick coronal fragments with razor blades and the aid of rat brain matrix. Obtained fragments transferred to optimum cutting temperature (OCT)-filled moulds were snap-frozen in liquid nitrogen and stored frozen until further analysis. Next the OTC embedded tissue was cryo-sectioned (Bright Instruments, UK) to 25 µm thick coronal slices subsequently collected on eight Menzel-Gläser Superfrost Plus slides (a slice collected every 200 µm) and stored in -20°C until staining. Once dried (45°C for an hour) and briefly washed with 50% ethanol, sections were outlined with a hydrophobic marker (PAP pen, Japan), rehydrated and blocked with 10% donkey serum in blocking solution (0.03% NaN_3, 0.3% Triton X-100 in PBS, pH=7.5) for two hours prior to overnight incubation at room temperature with primary antibodies against dopamine-β-hydroxylase (DBH, a marker of noradrenergic neurons: Mouse, 1:500, Millipore, MAB308, UK), mCherry (Rabbit, 1:500, Abcam, ab167453, UK). Slides were then PBS washed and incubated with the appropriate

fluorophore-conjugated secondary antibodies in blocking solution (Donkey anti-Rabbit, Alexa Fluor 568, A10042, Invitrogen, Eugene, OR, US; Donkey anti-Mouse, AlexaFluor 488, A21202, Invitrogen, Eugene, OR, US; all used at 1:1000 dilution) for 4 hours to overnight at room temperature. Slides were protected with mounting media (Fluoromount-G with DAPI, eBioscience, UK) and coverslips and stored in darkness at 4°C until imaging.

Samples were typically imaged with an LSM 710 laser-scanning confocal microscope (Zeiss) using Zeiss Plan Achromat 10x (0.3 NA) and 20 x (0.8 NA) dry objectives and analysed with Fiji Win 64. For quantification, samples were imaged with 20x dry objective on Zeiss Imager Z1 microscope coupled with AxioCam MRm CCD camera. The acquisition of images was made in multidimensional mode and the MosaiX function was used to construct the full view. 6-8 slices were imaged per animal. Cell counting was carried out on the Fiji Win 64 utilising cell counter plugin. On average, 20-30 brainstem sections were imaged for quantification.

Passive Tissue Clearing (PACT)

A passive CLARITY tissue clearing technique (PACT) (described in detailed[28]) has been implemented to allow thick (1000-2500 µm) spinal cord fragments imaging. Briefly, following transcardial perfusion of deeply anaesthetised rats with a cold PBS and a cold 4% PFA in phosphate buffer, pH=7.5, spinal cords were extracted and post-fixed in 4% PFA for 3-4 days in 4°C. After fixation, samples were transferred directly to ice-cold A4P0 solution consisting of: 4% acrylamide monomer (40% acrylamide solution, cat. 161-0140, Bio-Rad, UK), 0.25% VA-044 (thermoinitiator, Wako, US) in 0.01 M PBS, pH=7.4, and incubated at 4°C overnight in distilled water prewashed (to remove anticoagulant) vacutainer tubes (Vacutainer, #454087, Greiner GmbH, Austria). The next day, samples were degassed by piercing the septum with a 20G needle connected to a custom-build vacuum line. The residual oxygen was replaced with nitrogen by 2 min bubbling of the solution with pure nitrogen (BOC, UK) via a long, bottom-reaching 20G needle, and a second short needle pierced to allow gases to exhaust. Throughout, samples were kept on ice to prevent heating and consequent premature A4P0 polymerisation. After achieving oxygen-free conditions, samples were polymerised by 3 h incubation in a 37°C water bath. Following polymerisation, the excess honey-like polyacrylamide gel was removed with tissue paper, and samples were transferred to 50 ml falcon tubes filled with clearing solution. 10% SDS (#L3771, Sigma-Aldrich, UK) in PBS, pH=8.0, was used for passive

clearing. Samples were incubated on a rotary shaker at 37°C and 75 rpm (Phoenix Instruments, UK) until reaching the appropriate transparency (usually 3-4 days).

Next, all samples were washed extensively with PBS, pH=7.5, on rotary shaker at room temperature, by replacing the solution 4-5 times throughout the course of 1 day in order to remove the SDS. Following washing, samples were treated with primary antibody in blocking buffer consisting of 2% normal donkey serum in 0.1% Triton X-100 in PBS, pH=7.5 with 0.03% sodium azide. 400 μl of rabbit anti-tyrosine hydroxylase (TH, marker of catecholaminergic neurons; 1:250, Ab152, Millipore, UK) primary antibody was used per 2 mm thick spinal cord slice in a 2 ml Eppendorf tube. Samples were incubated with the primary antibody at room temperature, with gentle shaking for 3 days. This was followed by 4-5 washing steps with PBS over the course of a day days. Next, the samples were incubated with gentle agitation, at room temperature, in darkness, for 3 days with the goat anti-rabbit (Alexa Fluor 647, A21244, Invitrogen, Eugene, OR, US) fluorophore-conjugated secondary antibody (1:200 in the blocking buffer). Thereafter, samples were washed extensively with PBS at least 5 times over 1-2 days at room temperature. Finally, samples were overnight incubated in the refractive index-matching solution (RIMS, refractive index = 1.47) consisting of 40 g of Histodenz (#D2158, Sigma-Aldrich, UK) dissolved in 30 ml of PBS, pH=7.5 with 0.03% sodium azide. 400-600 μl of RIMS was used per structure. Samples were placed in fresh RIMS in custom-made glass slide chambers, covered with coverslips, and equilibrated for few hours before imaging.

Samples were imaged with a Zeiss LSM 780 one-photon confocal upright microscope, equipped with EC Plan-Neofluar 10x 0.3 NA, Ph1 dry objective (WD=5.3 mm, cat. 420341-9911, Zeiss, Germany) and 633 nm laser lines. Scans were taken with 2048x2048 pixel resolution, with 4-5 μm optical section typically spanning 400-700 μm of scanned depth (resulting in 100-150 planes) with auto Z-brightness correction to ensure uniform signal intensity throughout the sample. Images were exported from Zen 2012 Blue Edition software (Carl Zeiss Microscopy GmbH, Germany). Next graphical representations, 3D-rendering, animations, maximal intensity projections within selected z-stacks and further analysis were obtained with open-source Fiji (ImageJ) equipped with appropriate plugins.

Quantification and Statistical Analysis

All data plotted in represent mean ± SEM. Typically, up to 4 WDR neurons were characterised per preparation (n), and data were collected from at least 6 rats per group (N). Single pharmacological investigation was performed on one neuron per animal. Statistical analysis was performed either on number of neurons (n) for populational studies or number of animals (N) for pharmacological studies. Therefore, throughout the manuscript “n” refers to the number of cells tested and “N” to the number of animals tested. Detailed description of the number of samples analysed and their meanings, together with values obtained from statistical tests, can be found in each figure legend. Symbols denoting statistically significant differences were also explained in each figure legend. Main effects from analysis of variance (ANOVA) are expressed as an F-statistic and *p*-value within brackets. Throughout, a p-value below 0.05 was considered significant. One-way ANOVA with Tukey post-hoc test was used to assess significance for labelling efficiency between structures. Uncorrected two-way repeated-measures (RM) ANOVA with the Tukey post-hoc was used to assess von Frey and DNIC responses in the baseline conditions. For pharmacological experiments, Geisser-Greenhouse correction was used for RM-ANOVA. Paired student t-test was used to assess brush-evoked responses. GraphPad Prism was used to analyse the data.

Additional references:

1. Häring M, Zeisel A, Hochgerner H, et al. Neuronal atlas of the dorsal horn defines its architecture and links sensory input to transcriptional cell types. *Nat Neurosci*. 2018;21(6):869-880. doi:10.1038/s41593-018-0141-1

2. Millan MJ. Descending control of pain. *Prog Neurobiol*. 2002;66(6):355-474. doi:10.1016/S0301-0082(02)00009-6

3. Usoskin D, Furlan A, Islam S, et al. Unbiased classification of sensory neuron types by large-scale single-cell RNA sequencing. *Nat Neurosci*. 2014;18(1):145-153. doi:10.1038/nn.3881

4. Kohro Y, Matsuda T, Yoshihara K, et al. Spinal astrocytes in superficial laminae gate brainstem descending control of mechanosensory hypersensitivity. *Nat Neurosci*. 2020;23(11):1376-1387. doi:10.1038/s41593-020-00713-4

5. Olave MJ, Maxwell DJ. Axon terminals possessing the alpha 2c-adrenergic receptor in the rat dorsal horn are predominantly excitatory. *Brain Res*. 2003;965(1-2):269-273. doi:10.1016/S0006-8993(02)04124-0

6. Olave MJ, Maxwell DJ. Axon terminals possessing α2C-adrenergic receptors densely innervate neurons in the rat lateral spinal nucleus which respond to noxious stimulation. *Neuroscience*. 2004;126(2):391-403. doi:10.1016/j.neuroscience.2004.03.049

7. Olave MJ, Maxwell DJ. Neurokinin-1 projection cells in the rat dorsal horn receive synaptic contacts from axons that possess alpha2C-adrenergic receptors. *J Neurosci*. 2003;23(17):6837-6846.

8. Stone LS, Broberger C, Vulchanova L, et al. Differential distribution of alpha2A and alpha2C adrenergic receptor immunoreactivity in the rat spinal cord. *J Neurosci*. 1998;18(15):5928-5937.

9. Jensen BC, Swigart PM, Simpson PC. Ten commercial antibodies for alpha-1-adrenergic receptor subtypes are nonspecific. *Naunyn Schmiedebergs Arch Pharmacol*. 2009;379(4):409-412. doi:10.1007/s00210-008-0368-6

10. Zimmermann M. Ethical guidelines for investigations of experimental pain in conscious animals. *Pain*. 1983;16(2):109-110. doi:10.1016/0304-3959(83)90201-4

11. Kilkenny C, Browne WJ, Cuthill IC, Emerson M, Altman DG. Improving bioscience research reporting: the ARRIVE guidelines for reporting animal research. *PLoS Biol*. 2010;8(6):e1000412. doi:10.1371/journal.pbio.1000412

12. Szot P, Miguelez C, White SS, et al. A comprehensive analysis of the effect of DSP4 on the locus coeruleus noradrenergic system in the rat. *Neuroscience*. 2010;166(1):279-291. doi:10.1016/j.neuroscience.2009.12.027

13. Ross SB, Stenfors C. DSP4, a Selective Neurotoxin for the Locus Coeruleus Noradrenergic System. A Review of Its Mode of Action. *Neurotox Res*. 2015;27(1):15-30. doi:10.1007/s12640-014-9482-z

14. Vila-Pueyo M, Strother LC, Kefel M, Goadsby PJ, Holland PR. Divergent influences of the locus coeruleus on migraine pathophysiology. *Pain*. 2019;160(2):385-394. doi:10.1097/j.pain.0000000000001421

15. Hickey L, Li Y, Fyson SJ, et al. Optoactivation of Locus Ceruleus Neurons Evokes Bidirectional Changes in Thermal Nociception in Rats. *J Neurosci*. 2014;34(12):4148-4160. doi:10.1523/JNEUROSCI.4835-13.2014

16. Li Y, Hickey L, Perrins R, et al. Retrograde optogenetic characterization of the pontospinal module of the locus coeruleus with a canine adenoviral vector. *Brain Res*. 2016;1641:274-290. doi:10.1016/j.brainres.2016.02.023

17. Hirschberg S, Li Y, Randall A, Kremer EJ, Pickering AE. Functional dichotomy in spinal-vs prefrontal-projecting locus coeruleus modules splits descending noradrenergic analgesia from ascending aversion and anxiety in rats. *Elife*. 2017;6(Lc):1-26. doi:10.7554/eLife.29808.001

18. Wissing C, Maheu M, Wiegert S, Dieter A. Targeting Noradrenergic Neurons of the Locus Coeruleus : A Comparison of Model Systems and Strategies. *bioRxiv*. 2022;January 23:1-24. doi:10.1101/2022.01.22.477348

19. Urch CE, Dickenson a. H. In vivo single unit extracellular recordings from spinal cord neurones of rats. *Brain Res Protoc*. 2003;12:26-34. doi:10.1016/S1385-299X(03)00068-0

20. Bannister K, Patel R, Goncalves L, Townson L, Dickenson AH. Diffuse noxious inhibitory controls and nerve injury: restoring an imbalance between descending monoamine inhibitions and facilitations. *Pain*. 2015;156(9):1803-1811. doi:10.1097/j.pain.0000000000000240

21. Pertovaara A, Haapalinna A, Sirviö J, Virtanen R. Pharmacological properties, central nervous system effects, and potential therapeutic applications of atipamezole, a selective α2- adrenoceptor antagonist. *CNS Drug Rev*. 2005;11(3):273-288. https://www.scopus.com/inward/record.uri?eid=2-s2.0-30744477078&partnerID=40&md5=907bd567b59c4deb48a84ee16fa015e7

22. Graham RM. Selective alpha 1-adrenergic antagonists: therapeutically relevant antihypertensive agents. *Am J Cardiol*. 1984;53(3):16A-20A. doi:10.1016/0002-9149(84)90829-4

23. Rahman W, D'Mello R, Dickenson AH. Peripheral nerve injury-induced changes in spinal alpha(2)-adrenoceptor-mediated modulation of mechanically evoked dorsal horn neuronal responses. *J Pain*. 2008;9(4):350-359. doi:10.1016/j.jpain.2007.11.010

24. Bannister K, Lockwood S, Goncalves L, Patel R, Dickenson AHH. An investigation into the inhibitory function of serotonin in diffuse noxious inhibitory controls in the neuropathic rat. *Eur J Pain (United Kingdom)*. 2017;21(4):750-760. doi:10.1002/ejp.979

25. Hughes SW, Hickey L, Hulse RP, Lumb BM, Pickering AE. Endogenous analgesic action of the pontospinal noradrenergic system spatially restricts and temporally delays the progression of neuropathic pain following tibial nerve injury. *Pain*. 2013;154(9):1680-1690. doi:10.1016/j.pain.2013.05.010

26. Yaksh TL. Pharmacology of spinal adrenergic systems which modulate spinal nociceptive processing. *Pharmacol Biochem Behav*. 1985;22(5):845-858. doi:10.1016/0091-3057(85)90537-4

27. LeChasseur Y, Dufour S, Lavertu G, et al. A microprobe for parallel optical and electrical recordings from single neurons in vivo SUPPLEMENT. *Nat Methods*. 2011;8(4):319-325. doi:10.1038/nmeth.1572

28. Treweek JB, Chan KY, Flytzanis NC, et al. Whole-body tissue stabilization and selective extractions via tissue-hydrogel hybrids for high-resolution intact circuit mapping and phenotyping. *Nat Protoc*. 2015;10(11):1860-1896. doi:10.1038/nprot.2015.122
